# Impact of Tuberculosis Preventive Treatment on Adverse Pregnancy Outcomes in women living with HIV in Uganda: A Quasi-experimental study using routine care data

**DOI:** 10.21203/rs.3.rs-7170241/v1

**Published:** 2025-09-03

**Authors:** Joseph Musaazi, Christine Sekaggya-Wiltshire, Stella Zawedde-Muyanja, Proscovia M. Namuwenge, M. Sanni Ali, Yukari C Manabe, Barbara Castelnuovo, Nele Brusselaers

**Affiliations:** Infectious Diseases Institute, Makerere University College of Health Sciences; Infectious Diseases Institute, Makerere University College of Health Sciences; Infectious Diseases Institute, Makerere University College of Health Sciences; National Tuberculosis and Leprosy Program, Uganda Ministry of Health; London School of Hygiene & Tropical Medicine; Johns Hopkins University School of Medicine; Infectious Diseases Institute, Makerere University College of Health Sciences; University of Antwerp

**Keywords:** Tuberculosis Preventive Treatment (TPT), Pregnancy Outcomes, Women Living with HIV (WLHIV), Isoniazid Preventive Therapy (IPT), Sub-Saharan Africa, Routine Programmatic Data

## Abstract

**Background:**

The World Health Organization recommends tuberculosis preventive treatment (TPT) for people living with HIV, including pregnant women. However, data on the safety of TPT during pregnancy particularly from routine care settings in high tuberculosis (TB) burden countries remain limited. We evaluated the association between TPT exposure and adverse pregnancy outcomes among pregnant women living with HIV (WLHIV) in Uganda.

**Methods:**

We conducted a quasi-experimental study using routinely collected data from five public urban primary health care facilities in Kampala, Uganda. We included pregnant WLHIV on antiretroviral therapy (ART) between 2016 and 2023. The primary outcome was a composite of adverse pregnancy outcomes: miscarriage, stillbirth, low birth weight, congenital anomalies, or maternal/neonatal death. The primary exposure was 6-months isoniazid TPT (IPT) during pregnancy. Analyses used inverse probability of treatment weighting (IPTW) using logistic regression model to adjust for confounding and multiple imputation for handling missing data.

**Results:**

Analysis included 521 pregnant WLHIV, 44% were exposed to IPT during pregnancy. Overall, 10.0% experienced an adverse pregnancy outcome, with no significant difference between IPT-exposed and unexposed groups (10.3% vs. 9.6%; p = 0.81). Adjusted IPTW analysis showed no significant association between IPT exposure and adverse outcomes (pooled weighted odds ratio 1.04; 95% CI: 0.69–1.58). Sensitivity and subgroup analyses yielded consistent results.

**Conclusion:**

We found no evidence that 6-month isoniazid TPT increases the risk of adverse pregnancy outcomes. However, limitations in outcome and adverse event documentation from routine care may affect these findings. Strengthening pharmacovigilance and clinical reporting is essential to safeguard maternal and neonatal health as TPT coverage expands in high TB/HIV burden settings.

## INTRODUCTION

The World Health Organization (WHO) recommends tuberculosis preventive treatment (TPT) for people living with HIV (PLHV) including pregnant women, to reduce the risk of developing active tuberculosis(1). However, some studies show that pregnancy is associated with increased risk of hepatoxicity in women living with HIV(2), with higher risk among those exposed to isoniazid preventive therapy (IPT) compared to those not exposed(3). For instance, the IMPAACT P1078 randomized controlled trial, conducted across 13 sites in 8 high TB-burden countries reported a higher risk of adverse pregnancy outcomes in a composite outcome that included stillbirth or spontaneous abortion, low birth weight, preterm delivery, and congenital anomalies among women who initiated IPT during pregnancy (24%) versus those who deferred initiation until postpartum (17%) (4). Conversely, a systematic review that included the IMPAACT P1078 trial reported mixed findings. Of the five studies reviewed, only the P1078 trial, a randomized controlled trial, identified a significantly increased risk of adverse pregnancy outcomes associated with IPT exposure during pregnancy, whereas the remaining observational studies found no such association (3). These divergent findings highlight ongoing uncertainty about the safety of IPT during pregnancy, particularly in real-world settings.

Uganda bears a high burden of both TB and HIV. According to the 2023 joint United Nations programme on HIV/AIDS (UNAIDS), HIV prevalence among Ugandan women aged 15–49 years is 6.6%, nearly double that of men (3.6%) (5). The 2015 Uganda National TB Survey estimated TB incidence at 198 cases per 100,000 population (6). TB during pregnancy or the postpartum period has been associated with serious outcomes, including spontaneous abortion, low birth weight, preterm delivery (7), and perinatal mortality(8). Following WHO recommendations, the Uganda Ministry of Health (MoH) adopted TPT for high-risk populations in 2021, including pregnant women living with HIV (WLHIV), based on the presumption that benefits outweigh potential risks (9, 10). By 2023, TPT uptake and completion rates among PLHIV in Uganda had surpassed 90%, with higher completion observed among women than men (11, 12), reflecting both strong program implementation and national commitment to TB control.

Despite these gains, data on TPT safety during pregnancy remain limited, especially from routine care settings in high-burden countries. While both clinical trials and observational studies have assessed safety (3, 4, 13, 14), evidence from real-world programmatic data remains scarce. Although the P1078 trial demonstrated a modest increase in adverse outcomes with pregnancy-initiated IPT, WHO and Uganda-MoH recommends TPT for pregnant WLHIV especially residing in high TB burden settings, emphasizing that the benefits outweigh the risks.

This study aimed to evaluate the association between the tuberculosis preventive therapy (IPT) use during pregnancy and adverse pregnancy outcomes including spontaneous abortion or miscarriage, stillbirth, low birth weight, congenital anomalies, and maternal or neonatal death among pregnant WLHIV. Using routinely collected programmatic data, we assessed both the prevalence of adverse outcomes and the potential impact of TPT exposure during pregnancy.

## METHODS

### Study design, population and setting

#### Study design and population

We conducted a quasi-experimental study using routinely collected clinical data from five public primary health care facilities in Kampala Capital City Authority, Uganda. The clinics provide free care and treatment for people living with HIV (PLHV) through the President’s Emergency Plan for AIDS Relief (PEPFAR) program, including antiretroviral treatment, and prophylaxis for opportunistic infections and tuberculosis, and antenatal services. The clinics maintain registers for the TPT program, antenatal and post-partum visits; other individual clinical data is collected into the Uganda electronic medical record (EMR) system.

The study population included pregnant women living with HIV (WLHIV), aged 15 years and older, who initiated antiretroviral therapy (ART) between January 2016 and December 2023, coinciding with the rollout of the HIV Test and Treat strategy and expanded TPT provision in Uganda(15).

We excluded women with missing ART initiation dates, those with a ≥ 12-month gap in HIV clinic attendance, and women who initiated TPT before starting ART to minimize confounding due to the absence of ART’s protective effect against TB at the time of TPT initiation.

The study sites were purposively selected due to the relative completeness and reliability of their clinical documentation compared to other facilities in the region. Kampala was selected as the study setting due to its high burden of both HIV and tuberculosis (TB) relative to other regions in Uganda.

#### Programmatic Implementation of TPT in Uganda

In 2015, the Uganda-MoH adopted and expanded the WHO recommendation to provide TPT to all people at risk of developing active TB. This includes all PLHV including pregnant women without active TB, and household contacts of individuals with confirmed TB disease.

From 2015 until 2021, isoniazid monotherapy, taken daily for six months, was the only available TPT option recommended for asymptomatic PLHIV in Uganda(16). In 2021, the Uganda-MoH rolled out revised national TPT guidelines, incorporating rifamycin-based shorter-course regimens to improve uptake and adherence (9).

Between 2018 and 2023, the Uganda-MoH intensified efforts to expand TPT coverage among PLHV through a series of strategic interventions. In July 2019, the Uganda-MoH launched a 100-day accelerated IPT scale-up campaign aimed at initiating 300,000 PLHV on TPT and achieve > 90% completion rate among those initiated(17). In 2021, the Uganda-MoH updated national guidelines that integrated TPT into the routine HIV care package for all PLHV without symptoms of TB (9). These guidelines endorsed the use of shorter-course, rifamycin-based TPT regimens alongside isoniazid monotherapy, to improve adherence and programmatic uptake. While earlier TPT guidelines in Uganda permitted isoniazid use at any stage of pregnancy, the 2021 revision introduced more restrictive criteria for TPT provision in pregnant WLHV. Under the new guidance, TPT during pregnancy was recommended only for those with CD4 counts < 200 cells/mm^3^, WHO stage III or IV HIV disease, or a history of contact with a person with bacteriologically confirmed pulmonary TB. For all other pregnant WLHIV, TPT initiation was advised to be deferred until three months postpartum (9).

#### Data extraction and collection

Between July 2020 to March 2021, trained research assistants abstracted demographic, clinical, and pregnancy data from TPT, maternity and antenatal registers using a structured abstraction tool programmed in Open Data Kit (ODK)(18). To ensure data quality, built-in validation checks were applied within the ODK tool. Additional quality assurance measures included training research assistants in standardized data abstraction procedures and conducting daily reviews of the collected data.

Additional data were obtained directly from the Uganda Electronic medical records (EMR) database system (19) using a pre-defined abstraction guide. Data from the different sources were linked using the patient’s unique HIV clinic identifier number.

The specific data variables extracted are described in section below. To select study participants, systematic random sampling was employed at each of the five health facilities. The sampling interval was calculated by dividing the total number of eligible patients at each facility by the number of participants allocated to that site.

#### Outcome measure and covariates

The primary outcome was a composite measure of adverse pregnancy outcomes, defined as the occurrence of any of the following: spontaneous abortion/miscarriage, stillbirth, low birth weight, congenital anomalies, neonatal death, or maternal death. Individual pregnancy outcomes were assessed as secondary outcomes. The denominator was all pregnant WLHIV included in the study. For participants with multiple pregnancy events, the most recent pregnancy was used for analysis. The pregnancy event date was estimated as the first clinic visit at which pregnancy was recorded. Preterm birth was excluded from the composite outcome due to a lack of documented data in the reviewed pregnancy records.

### Definitions of individual pregnancy outcomes

#### Spontaneous Abortion (miscarriage)

The loss of a fetus before 20 gestation weeks of pregnancy.

#### Stillbirth

The death of a fetus at or after 20 weeks of pregnancy.

#### Low Birth Weight

Babies born weighing less than 2,500 grams (5 pounds, 8 ounces).

#### Congenital Anomalies in an Infant

Also known as birth defects, these are structural or functional abnormalities that occur during intrauterine life and can be identified prenatally, at birth, or later in life. Major anomalies to be defined according to the Metropolitan Atlanta Congenital Defects Program of the Centers for Disease Control and Prevention(27).

#### Neonatal Mortality

The death of a live-born baby within the first 28 days of life.

#### Maternal Mortality

The death of a woman during pregnancy, childbirth, or within 42 days of termination of pregnancy, from any cause related to or aggravated by the pregnancy or its management.

The primary exposure was tuberculosis preventive therapy (TPT) using 6-months isoniazid prophylaxis during pregnancy, which was the only regimen available during the study period. A secondary analysis further classified TPT exposure by trimester: no exposure, 1st trimester (< 13 weeks), 2nd trimester (13–28 weeks), and 3rd trimester (29–40 weeks), in accordance with national guidelines (20) and previous studies (4, 14).

Potential covariates included both clinical and reproductive factors. Clinical variables comprised viral load (copies/mL), nadir CD4 count at ART start (cells/μL), ART regimen type at the time of pregnancy, ART duration in months, WHO HIV clinical stage (I/II vs III/IV), body mass index (BMI, kg/m^2^) and, ART initiation calendar year before and after September 2018 to coincides with dolutegravir-based ART regimen roll-out in Uganda. Viral load during pregnancy was defined as the closest measurement within 12 months before or 6 months after the pregnancy event date. Baseline CD4 count at ART start was defined as any test conducted within 6 months before or 3 months after ART start. Reproductive factors included maternal age (in years), gestational age (in weeks) at first antenatal care (ANC) visit, parity, gravidity, number of ANC visits, and anemia. Information on other maternal health conditions such as preeclampsia, hypertension, diabetes, and sexually transmitted infections (including syphilis) were limited or inconsistently recorded and therefore excluded from the analysis. Pre-pregnancy weight was defined as the most recent recorded weight within six months prior to the first documented pregnancy visit.

#### Sample size and statistical power

A total sample size of 538 pregnant WLHIV (269 per group) was estimated to provide 80% power to detect a ≥ 10 percentage-point difference in adverse pregnancy outcomes between exposed and unexposed groups, assuming a baseline risk of 17% in the unexposed group (4), with a two-sided alpha of 0.05 and a Pearson Chi-square test.

### Statistical analysis

Descriptive statistics (frequencies, percentages, means with standard deviations, or medians with interquartile ranges) were used to summarize participant characteristics. For longitudinal variables such as weight, missing values were imputed using last observation carried forward (LOCF).

The primary outcome; any adverse pregnancy outcome was compared between women exposed and unexposed to TPT during pregnancy using the Pearson Chi-square test. To address confounding, inverse probability of treatment weighting (IPTW) using stabilized propensity scores was applied, followed by weighted logistic regression models for effect estimation. Missing data were addressed using multiple imputation by chained equations (MICE) with five imputations via predictive mean matching for continuous and logistic model for binary variables. Details of the imputation models used for each covariate with missing values are provided in Supplementary Section, Table S1.

Subgroup analyses were conducted based on CD4 count (< 200 vs ≥ 200 cells/μL), viral load (< 200 vs ≥ 200 copies/mL), age (< 25 vs ≥ 25 years), ART initiation year (< 2018 vs ≥ 2018), and BMI (< 18.5, 18.5–<25, ≥ 25 kg/m^2^), selected based on clinical relevance and prior evidence. Pregnancy weight was defined as the most recent weight within six months prior to the first recorded pregnancy visit.

Sensitivity analyses included unadjusted, covariate-adjusted, and Firth penalized logistic regression models, the latter used to address sparse outcomes. Firth regression was not used in the primary analysis due to incompatibility with imputed datasets. Statistical significance was set at 5% level.

## RESULTS

### Participants description

A total of 521 pregnant women living with HIV were included in this analysis (Table 1). Of these, 227 (44%) had used IPT for TPT during pregnancy, while 294 (56%) had not. Among those who used IPT, 139 (61%) started IPT in 1st trimester, 58 (26%) in the 2nd trimester, and 30 (13%) in the 3rd trimester. In the IPT unexposed group, 149 (51%) had completed IPT course before the onset of pregnancy, and 145 (49%) had never received IPT.

Overall, the median age at current pregnancy was 28 years (interquartile range [IQR] 24 to 31 years), 50.3% (238/473) had ≥ 2 prior live births, 0.6% were in HIV WHO stage 3 or 4, with median time on ART of 22 months (IQR: 0 to 53 months). At their first recorded antenatal visits, the median gestation age was 24 weeks (IQR: 18 to 30 weeks), implying that majority of the women had their first ANC visit in their 2nd trimester (Table 1).

### Pregnancy outcomes

Of the 521 enrolled participants, pregnancy outcome data were available for 472 (90.6%) (Table 2). Among those with complete data, 47 women (10.0%) experienced at least one adverse pregnancy outcome.

There was no statistically significant difference in the prevalence of adverse pregnancy outcomes between women exposed to TPT during pregnancy and those unexposed (22/213, 10.3% versus 25/259, 9.6% respectively, p-value = 0.81). After adjusting for potential confounding using inverse probability of treatment weighting (IPTW), and completing missing values using multiple imputation, the pooled weighted estimates still showed no evidence for an increased risk of adverse pregnancy outcome due to TPT exposure during pregnancy (pooled weighted odds ratio [OR], 1.04; 95% confidence interval [CI], 0.69 to 1.58; p = 0.84) (Table 3). The distribution of composite adverse pregnancy outcomes across categories of covariates is detailed in Supplementary Section, Table S2.

Sensitivity analyses yielded consistent results; re-running the analysis using Firth penalized logistic regression which accounts for sparse data bias, it further revealed no effect of TPT exposure during pregnancy on risk of adverse pregnancy outcome (adjusted OR, 1.08; 95% CI, 0.59 to 1.97). Similarly, conventional complete case analysis and covariate-adjusted logistic regression with multiply-imputed data showed no significant association (Table 3).

**Figure 1:**
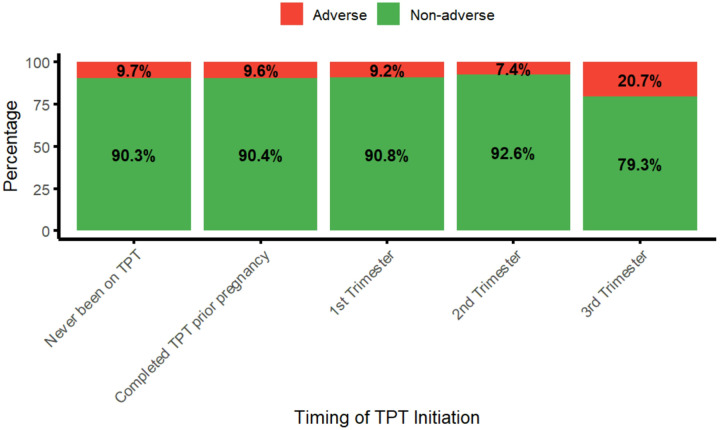
Prevalence of adverse pregnancy outcomes by timing of TPT exposure

Low birth weight was the most frequent adverse outcome, occurring in 16 of 472 participants (3.4%), but was not significantly different between the TPT-exposed and TPT-unexposed (p-value = 0.53). The overall median birth weight was 3200 g (IQR, 3000 to 3500 g). Additionally, there was no significant association between TPT exposure and other individual pregnancy outcomes; spontaneous abortion, stillbirth, neonatal or maternal deaths (all p-values were ≥ 0.20) (Table 2, Fig. 1).

Among women who received TPT during pregnancy, the highest prevalence of adverse outcomes was observed in those who initiated TPT in the third trimester (6/29; 20.7%), compared with those who initiated in the first (12/130; 9.2%) or second trimester (4/54; 7.4%). However, this difference did not reach statistical significance (p-value = 0.38) (Fig. 1, Supplementary Section Table S3). Stillbirth was the most frequently observed adverse event among those who initiated TPT in the third trimester (Supplementary Section, Table S4).

**Figure 2:**
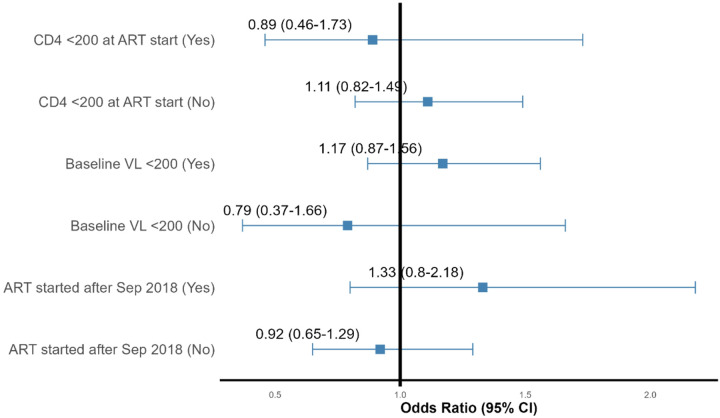
Subgroup Analysis for the impact of TPT-exposure during pregnancy on pregnancy outcome Subgroups for ART started after Sep 2018 (Yes) denotes participants initiated on ART after September 2018, which coincides with dolutegravir-based ART regimen roll-out in Uganda, whereas ART started after Sep 2018 (No) denotes those were initiated on ART before September 2018.

In exploratory subgroup analyses (Fig. 2), there was no significant interactions effect of being on TPT during pregnancy, and selected covariates which included; timing of ART initiation, HIV viral load at the first antenatal visit, or baseline CD4 count (all interaction p > 0.10).

Adverse pregnancy outcomes defined as proportion of composite outcome that includes any of the following: spontaneous abortion/miscarriage, stillbirth, preterm delivery, low birth weight, congenital anomalies, neonatal death, or maternal death.

## Discussion

This study contributes to the body of knowledge about safety of TPT during pregnancy. Using routinely collected programmatic data from urban HIV clinics in Uganda, we evaluated the association between TPT exposure during pregnancy and adverse pregnancy outcomes. We found no statistically significant increase in the risk of composite or individual adverse outcomes that included; spontaneous abortion or miscarriage, stillbirth, low birth weight, congenital anomalies, maternal, or neonatal death among WLHIV on antiretroviral therapy (ART) who received IPT. These findings were consistent across causal inference models and sensitivity analyses, supporting their robustness.

We did not observe evidence of effect modification by maternal viral load during pregnancy, baseline CD4 count, or timing of IPT initiation in relation to the rollout of dolutegravir-based ART in Uganda. However, the study may have been underpowered to detect interaction effects, limiting our ability to assess heterogeneity in IPT safety across subgroups.

Low birth weight emerged as the most common adverse outcome, consistent with findings from the IMPAACT P1078 trial, which attributed low birth weight to maternal factors such as poor nutrition and smoking(14). Unfortunately, our dataset lacked information on these potential confounders, limiting exploration of their role in our cohort.

Although statistically non-significant, we observed slightly higher proportions of stillbirth and congenital anomalies of about 2 percentage points higher among IPT-exposed women. Notably, four of the six stillbirths occurred among those initiating IPT in the first trimester, a period critical to fetal development (21). These signals underscore the need for continued pharmacovigilance and larger studies to assess the safety of IPT during early pregnancy (22).

We also noted a higher frequency of adverse outcomes among women initiating IPT in the third trimester. Late IPT initiation may reflect delayed antenatal care, a known risk factor for poor outcomes such as stillbirth and preterm birth (23, 24). These findings highlight the need to promote early antenatal attendance and ensure timely initiation of preventive therapies like IPT to improve pregnancy outcomes.

Our results align with observational studies from sub-Saharan Africa (13, 25, 26), and a systematic review by Hamada et al. (3), all suggesting no increased risk of adverse pregnancy outcomes with IPT. For instance, Taylor et al. (25) found no association between IPT and adverse outcomes in Botswana, while Salazar-Austin et al. (26) and Kalk et al. (13) reported lower risks of adverse outcomes among IPT recipients in cohorts in South Africa. However, these studies are subject to residual confounding, as are ours, due to limited adjustment for key maternal risk factors.

In contrast, Theron et al. (14), using data from the IMPAACT P1078 randomized trial, reported significantly higher rates of composite adverse pregnancy outcomes among women initiating IPT during pregnancy versus postpartum. That study had strong internal validity, with a large sample size (925 mother–infant pairs), conducted in 8 high TB countries, and rigorous adjustment for confounders. While our study used causal inference methods to minimize bias, unmeasured factors such as syphilis, hepatitis co-infection, multiple gestation, and other comorbidities were often undocumented and could still influence outcomes.

Absence of increased risk of adverse pregnancy outcomes may reflect lower baseline risk in our cohort, as all women were already on ART at conception, and achieved viral suppression during pregnancy, factors known to protect against adverse outcomes and potentially mitigate adverse pregnancy outcomes.

A major strength of this study is its use of routine electronic medical record (EMR) data from multiple high-burden HIV/TB clinics in Uganda, enhancing generalizability in similar programmatic settings. Such data allow for practical evaluation of intervention safety within routine care, informing policy in contexts where randomized trials may not be feasible.

Nonetheless, several limitations warrant consideration. First, misclassification and underreporting are possible, particularly for early pregnancy losses, preterm births, congenital anomalies, and maternal deaths, which may be inconsistently documented. Second, selection bias may have occurred, as most women initiated antenatal care in the second trimester, potentially excluding early losses. Third, key confounders such as syphilis, hepatitis, multiple gestation, nutritional status, and smoking were not available, limiting our ability to adjust for all relevant risk factors (14). Finally, our modest sample size limited power to detect small or rare effects, though linked EMR data across several clinics enhances the external validity of our findings.

## Conclusion

Our findings show no evidence that 6-month isoniazid monotherapy TPT, delivered through routine HIV and antenatal care in Uganda, increases the risk of adverse pregnancy outcomes. However, interpretation should consider limitations such as limited statistical power and incomplete outcome documentation inherent to routine programmatic data. As TPT scale-up continues especially among pregnant women in high TB/HIV burden settings strengthening pharmacovigilance systems and ensuring systematic documentation of adverse events and relevant risk factors is essential. These efforts are critical to safeguarding maternal and neonatal health while supporting national and global goals for TB prevention among vulnerable populations, including women living with HIV.

## Supplementary Material

This is a list of supplementary files associated with this preprint. Click to download.

- SUPPLEMENTARY.docx

## Figures and Tables

**Table 1:** Pregnant women living with HIV’ characteristics at first antenatal visit by TPT exposure status during pregnancy*

Characteristics	Total	TPT Unexposed group	TPT Exposed group	P-value
	N = 521	N = 294	N = 227	
Age in years, median (IQR) †	28 (24, 31)	28 (24, 32)	28 (24, 31)	0.403‡
Parity ≥ 2 births†	238 (50.3)	125 (48.4)	113 (52.6)	0.374
BMI in Kgs/m^2^, median (IQR) †	24.9 (22.3, 27.8)	24.9 (22.2, 27.8)	24.9 (22.7, 27.5)	0.750‡
WHO HIV stage 3/4, n (%) †	3 (0.6)	1 (0.4)	2 (0.9)	0.469
Gestation age at first ANC visit in weeks, median (IQR)	24 (18, 30)	23 (18, 30)	26 (20, 32)	0.013‡
CD4 count at ART start, in cells/mL, median (IQR) †	408 (243, 584)	413 (215, 586)	396 (268, 584)	0.820‡
Nadir CD4 count < 200 cells/mL, n (%)	73 (18.7)	45 (21.2)	28 (15.6)	0.158
Viral load at pregnancy †				
< 200 copies/mL, n (%)	299 (89.2)	163 (88.1)	136 (90.7)	0.452
< 1000 copies/mL, n (%)	308 (91.9)	168 (90.8)	140 (93.3)	0.444
ART start after 2018 †¥	142 (32.1)	50 (21.3)	92 (44.4)	<0.001
ART duration in months, median (IQR) †	22 (0, 53)	25 (6, 56)	16 (0, 46)	<0.001 ‡
ART duration ≥ 2 years, n (%) †	245 (48.9)	144 (52.6)	101 (44.5)	0.072
ART regimen at pregnancy ¶				
DTG based	33 (6.7)	21 (7.7)	12 (5.4)	0.559
EFV based	444 (89.5)	240 (87.9)	204 (91.5)	
NVP based	10 (2.0)	7 (2.6)	3 (1.3)	
ATV/r or LPV/r	9 (1.8)	5 (1.8)	4 (1.8)	

* Number of women exposed to TPT during pregnancy in: 1st trimester (n = 139), 2nd trimester (n = 58), and 3rd trimester (n = 30). Among the unexposed group, n = 149 had completed TPT before pregnancy onset, n = 145 had never been exposed to TPT.

† Missing values: age (n = 3), parity (n = 48), BMI (n = 38), WHO stage (n = 47), nadir CD4 (n = 130, 25%), Viral load (n = 186, 36%), ART duration (n = 20, 4%), 5 of those with ART start date had no regimen specified.

‡ P-values derived using Man-Whitney Wilcoxon rank-sum test to comparing medians across groups, else P-values were derived using Pearson Chi-square test

¥ ART initiation calendar year before and after September 2018 to coincides with dolutegravir-based ART regimen roll-out in Uganda. This stratification helped to explore whether there’s interaction between TPT exposure before versus after DTG-based regimen DTG roll-out in Uganda.

¶ ART regimen at pregnancy, 97% were on TDF based regimen on overall (96% in unexposed group, 98% in exposed group).

**Table 2:** Pregnancy outcomes by TPT-exposure status during pregnancy

Pregnancy outcomes¶	TPT-exposed n (%) N = 213	TPT-unexposed n (%) N = 259	Risk difference (%)	P-value†
Non-adverse outcome	191 (89.7)	234 (90.3)	N/A	0.81
Adverse outcome	22 (10.3)	25 (9.6)	0.7 (−4.8,6.1)	
*Specific adverse outcomes*				
Low birth weight (< 2500g)	6 (2.8)	10 (3.8)	−1.0 (−4.3, 2.2)	0.53
Spontaneous abortion or miscarriage	3 (1.4)	6 (2.3)	−0.9 (−3.3, 1.5)	0.47
Stillbirth	6 (2.8)	2 (0.7)	2.0 (−0.4, 4.5)	0.09
Neonatal death	0	1 (0.4)	−0.4 (−1.1, 0.4)	0.37
Maternal death	0	2 (0.7)	−0.8 (−1.8, 0.3)	0.20
Other anomalies: Difficulty in breathing	7 (3.3)	4 (1.5)	1.7 (−1.1, 4.6)	0.21

¶ Analysis performed on n = 472 women who had pregnancy outcomes, 49/521 (9%) had missing pregnancy outcome.

† Pearson Chi-square P-value comparing adverse pregnancy outcomes between women exposed to TPT during pregnancy versus those who were not. % denote column percentages

**Table 3:** Weighted odds ratios and 95% confidence intervals for the impact of Isoniazid TPT monotherapy taken during pregnancy on adverse pregnancy outcomes

	Odds Ratio (95% CI)	*P*-value
**Primary analysis**		
IPTW weighted Logistic regression model¥	1.04 (0.69, 1.58)	0.84
**Sensitivity analyses**		
Unadjusted Logistic regression model (complete case)¶	1.08 (0.59, 1.97)	0.81
Unadjusted Logistic regression model (imputed)	1.08 (0.60, 1.96)	0.80
Covariates-adjusted Logistic regression model^†^	1.01(0.51, 12.00)	0.97
Firth penalized logistic regression model^‡¶^	1.08 (0.59, 1.97)	0.81

OR = odds ratio; IPTW = inverse probability of treatment weighting; TPT = tuberculosis preventive therapy.

¥ Pooled Odds Ratios and their 95% confidence intervals (95%CI) were estimated from Pooled Weighted Logistic Regression. Propensity scores used to generate Inverse Probability Treatment Weighted (IPTW) weights were calculated using a logistic regression model with covariates: maternal age, body mass index, anemia during antenatal care, ART duration, CD4 < 200, viral load < 200, ART regimen timing (pre/post-September 2018), and parity ≥ 2. Missing data were completed using Multiple Imputation with chained equations using 5 imputations, via predictive mean matching for continuous and logistic model for binary variables. See Table S1 for details on specific imputation models used.

¶ Complete case estimates analyzed on 472 participants (excluding 49 with missing pregnancy outcomes)

† Covariate-adjusted logistic regression model included the same variables as the PS model.

‡ Firth penalized logistic regression model used to reduce sparse data bias.

## Data Availability

The datasets used and/or analysed during the current study are available from the corresponding author on reasonable request.

## Abbreviations

WHO: World Health Organization
HIV: Human Immunodeficiency Virus
AIDS: Acquired Immune Deficiency Syndrome
TB: Tuberculosis
TPT: Tuberculosis Preventive Treatment
IPT: Isoniazid Preventive Therapy
ART: Antiretroviral Therapy
PLHV: People Living with HIV
WLHIV: Women Living with HIV
Uganda: MoH-Uganda Ministry of Health
EMR: Electronic Medical Record
IPTW: Inverse Probability of Treatment Weighting
